# Prevalence of dental agenesis in a sample of Italian orthodontic patients: an epidemiological study

**DOI:** 10.1186/s40510-017-0186-9

**Published:** 2017-10-16

**Authors:** Antonio L. T. Gracco, Serena Zanatta, Filippo Forin Valvecchi, Denis Bignotti, Alessandro Perri, Francesco Baciliero

**Affiliations:** 1 Department of Neurosciences, Section of Dentistry, University of Padua, Via Giustiniani 2, Padua, Italy; 2 Vicenza, Italy

**Keywords:** Hypodontia, Prevalence, Dental agenesis

## Abstract

**Background:**

The congenital absence of one or more teeth is a dental anomaly that frequently occurs in the world’s population with a wide variability of distribution. The aim of this study is to assess the current prevalence of dental agenesis in the permanent dentition (excluding third molars) using a sample of Italian orthodontic patients.

**Methods:**

Panoramic radiographs of 4006 Caucasian children between 9 and 16 years of age (1865 males and 2141 females) performed over a 5-year period (from 2010 to 2015) were carefully examined to identify congenital missing teeth. A chi-square test was used to determine the difference in the prevalence of hypodontia between genders and between arches.

**Results:**

The prevalence of dental agenesis was 9% (9.1% for females and 8.7% for males). The most common congenitally missing teeth were the mandibular second premolars (20.3 and 18.1%) followed by the upper lateral incisors (17.8 and 17.7%) and the maxillary second premolars (7.4 and 6.3%). The absence of one tooth to five teeth was observed in 344 patients (8.6%), while 15 patients showed from six to nine missing teeth (0.4%). The analysis showed 363 cases of agenesis in the upper arch (0.64%) and 339 in the lower arch (0.60%). Unilateral (4.6%) and bilateral (4.4%) agenesis demonstrated a similar frequency. The most common bilateral missing teeth were the mandibular second premolars (1.9%) and the maxillary lateral incisors (1.6%).

**Conclusions:**

The results of this study observed a higher prevalence of hypodontia compared to previous studies conducted on the Italian population. Thus, a detailed and careful radiographic examination was important in diagnosing one or more missing teeth. This could help plan the best possible treatments, both esthetically and functionally, for these patients.

## Background

Tooth agenesis is considered one of the most common anomalies of dental development and occurs with a high frequency in the world’s population compared to other development abnormalities [1–8]. Permanent dentition (2.3 to 9.6%) is observed much more frequently than the primary (0.1–0.7%) [6, 9]. Congenital or developmental absence of one or more teeth has been described in literature with different terms [2, 3, 7, 9, 10]. Congenital absence of one to six teeth (excluding the third molars) is generally called “hypodontia,” while the absence of more than six teeth is named “oligodontia” and “anodontia”, a very rare condition, is the absence of all teeth.

Hypodontia may occur both as non-syndromic form as an isolated trait (familial agenesis) [9] and as a manifestation of a genetic syndrome [2, 4, 6]. More than 49 syndromes have been associated with one or more missing teeth [3, 6]; the main ones are hypohidrotic ectodermal dysplasia, incontinentia pigmenti, Down syndrome, craniofacial dysostosis, and syndromes associated with growth and development defects [2, 11].

Studies based on prevalence and distribution of hypodontia demonstrated a high variability depending on sample size, gender, race, and ethnic provenance [1, 8–10, 12, 13].

A meta-analysis conducted by Polder showed that prevalence varied in the world from 2.2% in the Saudi Arabia to 6.3% in the Australian population. In the European population, it varied from 3.4% in Switzerland to 10.1% in the population of Norway [10].

A Medline research project performed in June 2015 found five studies on the prevalence of hypodontia in the Italian population [14–18]. The most recent was published in 1993, so our purpose was to assess whether the prevalence of congenital missing teeth had changed compared to previous studies. Moreover, this is the first research project performed in Italy on a sample of orthodontic patients.

The purpose of this study was to examine the current prevalence and distribution of hypodontia in the permanent dentition (excluding third molars) in a sample of Italian orthodontic patients, determining which are the most affected teeth and to compare our results with those of other studies.

## Methods

This is a retrospective, observational, and multicentric study. The research was approved by the Ethics Committee of the Medical School of the University of Padua (protocol number 41648). All patients signed a formal consent for researchers to use and publish their personal data in the research project.

The initial sample included all panoramic radiographs of Italian orthodontic patients (4196) between 9 and 16 years of age performed over a 5-year period (2010–2015) at the Dental School of the University of Padua and two private dental offices located in Vicenza and Verona. All patients visited these dental clinics for an orthodontic evaluation. The radiographic machines were the same with uniform features. The inclusion criteria for this study were patients of Caucasian origin, patients with no history of medical problems, patients with no history of any syndrome, presence of panoramic radiograph with good quality, and patients between 9 and 16 years of age.

All selected files were examined by the same operator in a dark room using X-ray viewer to identify the presence of dental agenesis (excluding third molars). A tooth was diagnosed as congenitally missing if the mineralization of its crown could not be identified on orthopantomogram. The operator analyzed the records and the medical history of the patients and excluded 190 records, considering the following exclusion criteria: agenesis of third molars, patients with missing teeth for decay processes, avulsions or extracted for orthodontics or other reasons, panoramic radiography of Non-Caucasian patients, patients with facial clefts and craniofacial syndromes, and poor image quality of panoramic radiographs. The final sample of this study included 4006 panoramic radiographs: 1865 males with a mean age of 11.1 years and 2141 females with a mean age of 12.4 years. Data obtained from panoramic radiographs and patients’ records were recorded according to gender, subject’s date of birth, age at time of radiography, number of missing teeth and their location, maxillary versus mandibular agenesis, and right versus left side.

### Statistical analysis

The data was analyzed using the R software version 3.2.2 (R Core Team, 2015) on Linux/Ubuntu 12.04.

Data found in this study was described using descriptive statistical analysis. The chi-square statistical test was applied to analyze the frequency of agenesis between genders (males/females) and between maxilla and mandible; the level of significance was set at *P* < 0.05.

## Results

The final dataset comprised 4006 patients of Caucasian origin, of which 3647 had no missing permanent teeth. A total of 196 females and 163 males examined showed at least one congenitally missing tooth (excluding third molars), bringing the total to 359 patients. The female hypodontia prevalence was higher than males (9.1 and 8.7%, respectively), although difference between gender was not statistically significant. The overall prevalence of hypodontia was found to be 9% of the total sample population. The overall prevalence of oligodontia was 0.4% of the studied sample: there were 15 patients, nine females (0.22%) and six males (0.15%) with the absence of six or more teeth excluding the third molars (Table 1).

**Table 1:** Distribution of the patients by gender and number of missing teeth

	Number of missing teeth	Males	Females	Total
Patients with dental agenesis	From 1 to 5 (hypodontia)	157 (3.9%)	187 (4.7%)	344 (8.6%)
	6 or more (oligodontia)	9 (0.2%)	6 (0.1%)	15 (0.3%)
Patients without dental agenesis	No one	1702 (42.5%)	1945 (48.5%)	3647 (91%)
Total		1868 (46.6%)	2138 (53.3%)	4006 (100%)

**Fig. 1:**
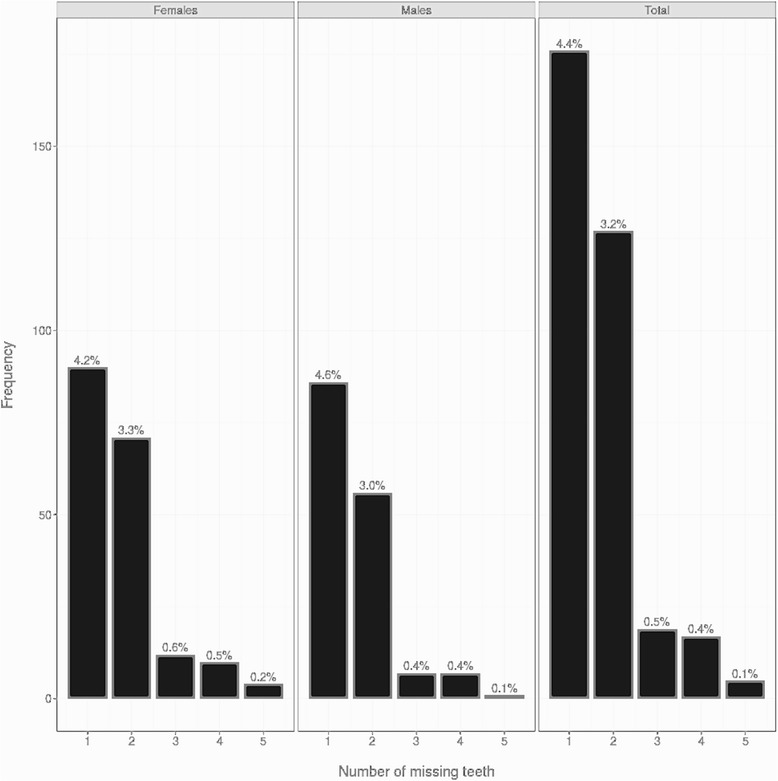
Frequencies and percentages of agenesis in males, in females, and in total sample

In 359 patients, a total of 702 permanent teeth were missing (401 in females [0.7%] and 301 in males [0.6%]). Of all the examined patients, 176 had one missing tooth (4.4%), 127 (3.2%) had two missing teeth, 19 (0.5%) had three missing teeth, 17 (0.4%) had four missing teeth, and 5 (0.1%) had five missing teeth. The difference between males and females was not statistically different (Fig. 1).

The most commonly congenitally missing teeth were the lower left second premolar (20.3% of the sample), followed by the lower right second premolar (18.1%), the upper lateral incisors (17.8 and 17.7%), the upper left second premolar (7.4%), the upper right second premolar (6.3%), and the upper right first premolar (2.6%). There were no significant differences between the right and left sides for any particular tooth (Table 2).

**Table 2:** Ranking of the most frequent missing teeth divided between sexes

Teeth	35	45	12	22	25	15	14	24	32	42	34	44	Total
Males *N* (%)	41 (7.8)	39 (7.4)	39 (7.4)	43 (8.2)	20 (3.8)	11 (2.1)	10 (1.9)	8 (1.5)	8 (1.5)	6 (1.1)	5 (0.9)	4 (0.8)	234 (44.5)
Females *N* (%)	66 (12.5)	56 (10.6)	55 (10.4)	50 (9.5)	19 (3.6)	22 (4.2)	4 (0.8)	5 (0.9)	3 (0.6)	5 (0.9)	4 (0.8)	3 (0.6)	292 (55.5)
Total *N* (%)	107 (20.3)	95 (18)	94 (17.8)	93 (17.7)	39 (7.4)	33 (6.3)	14 (2.6)	13 (2.5)	11 (2.1)	11 (2.1)	9 (1.7)	7 (1.3)	526 (100)

There were 363 missing teeth in the maxilla (0.64%) and 339 missing teeth in the mandible (0.60%). The number of missing teeth in the maxilla was slightly higher, but the difference between the two arches was not statistically significant (*χ* ^2^ (1) = 0.76, *P* = 0.38). There were 118 patients with one missing tooth in the upper arch (2.9%) while in the lower arch there were 90 (2.2%). In the upper arch, there were 90 patients with two missing teeth (2.02%), and in the lower arch, there were 84 (2.09%) (Table 3).

**Table 3:** Number of dental agenesis divided by dental arches

Number of dental agenesis per patient										
	0	1	2	3	4	5	6	7	8	9
Maxilla	3788 (94.5%)	118 (2.9%)	81 (2.02%)	8 (0.2%)	6 (0.14%)	1 (0.02%)	1 (0.02%)	1 (0.02%)	1 (0.02%)	1 (0.02%)
Mandible	3811 (95.1%)	90 (2.2%)	84 (2.09%)	11 (0.3%)	6 (0.14%)	2 (0.05%)	2 (0.05%)	0	0	0

There were at least 218 patients with at least one missing tooth in the maxilla (5.4%), while there were fewer patients missing at least one tooth in the mandible, equal to 195 (4.7%). The number of patients with agenesis did not differ statistically significantly between the maxilla and the mandible (*χ* ^2^ (1) = 1.23, *p* = 0.26).

Unilateral agenesis occurred with a frequency of 4.6%; bilateral agenesis manifested a frequency of 4.4%. Unilateral and bilateral agenesis had the same probability of occurrence, with no difference between genders: 4.4% of females had a unilateral agenesis and 4.8% a bilateral agenesis. Likewise, 4.7% of males showed a unilateral agenesis and 4.0% a bilateral agenesis. The chi-square test pointed out a similarity of males and females on this feature (*χ* ^2^ (2) = 1.49, *p* = 0.47).

Teeth that resulted missing simultaneously were mainly mandibular second premolars (1.9%) and the two maxillary lateral incisors (1.6%). This pairing of agenesis can also sometimes be found missing in cross combination: patients with four or more missing teeth presented the agenesis of mandibular second premolars combined with the two lateral incisors or with the maxillary second premolars.

**Fig. 2:**
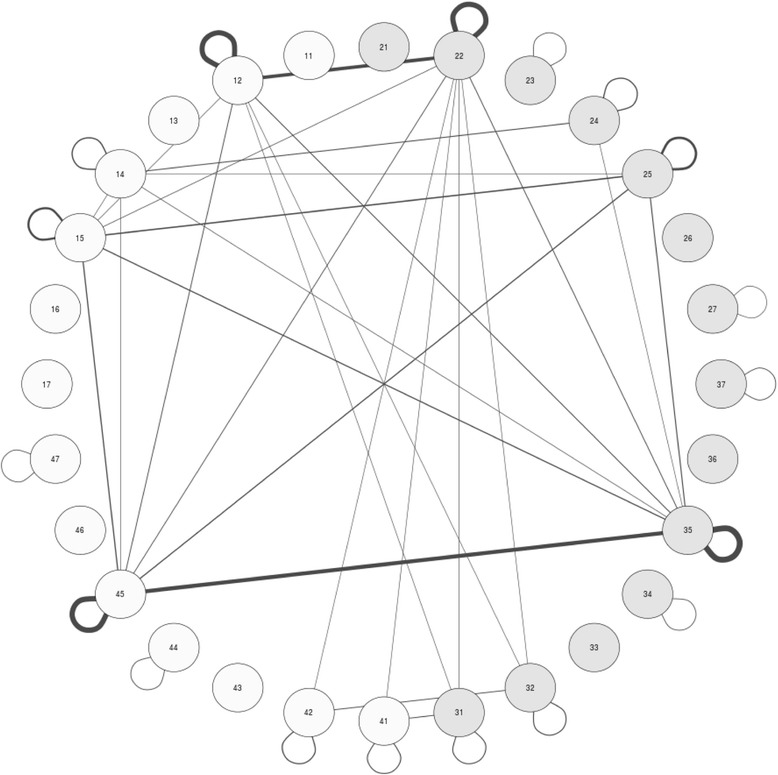
Network graph representing the two dental arches. Each tooth is represented by a node. Missing teeth in more than 5 patients are marked with a green ring: the thicker the ring is, the higher the frequency of agenesis for that tooth. Teeth which presented simultaneous agenesis are connected to each other: the thicker the line, the higher the frequency for that pair of teeth

We defined “co-occurrence” as the concomitant lack of two teeth; we calculated the patients in which each tooth was missing in conjunction with all others. We built a graph in which each node represented a tooth (Fig. 2).

## Discussion

The prevalence of tooth agenesis, excluding third molars, was observed at 4.89% among females and 4.07% for males, for a total of about 9% for both sexes together. This result showed a higher prevalence compared with the two previous Italian studies on this topic and confirms that hypodontia is a common developmental anomaly in Italian children. In the analysis of Lo Muzio et al., the prevalence was 5.17% [14], and according to the data of Polastri et al., the prevalence was 5.14% [15]. The sample studied by Polastri et al. included 700 national servicemen aged between 19 and 26, so it was much smaller and very different from our sample of patients. This research is the first of its kind in Italy analyzing the prevalence of dental agenesis in a sample of orthodontic patients. Comparing our results with those of a study of the non-orthodontic Italian sample, we found a slightly higher prevalence of dental agenesis (9% against 7.1%), perhaps because it is more likely that a patient with one or more missing teeth gets to an orthodontic visit.

According to literature review on the prevalence of agenesis, we could state that the range of prevalence values varies from 2.8% in the Malaysian population [19] to 12.6% in the German population [20].

Also, in the same population, different studies reported various values of prevalence: Celikoglu et al. determined prevalence of 4.6% in Turkish orthodontic patients [21] while Sisman et al. found a prevalence of 7.54% in another sample of the Turkish population [22].

The result of this study pointed out a higher prevalence in Italy than in most other countries. A higher prevalence rate was found in a few other studies: Chung et al. estimated a prevalence of 11.2% in Korean population [23] and Hunstadbraten of 10.1% in Norway [10]. A very high prevalence was also reported in two German studies (12.6% [20] and 11.3% [6]). The wide range of prevalence values observed in these studies has indicated that geographic, gender, races, and genetics differences as well as the big differences in the sample size and criteria of selection play a fundamental role in the varied results of studies of hypodontia. This wide range could make the comparison of the result of this study very limiting with other previous studies.

Polder examined a total of 28 studies and concluded that the prevalence of dental agenesis in females was almost 1.4 times higher than in males [10]. In this study, there was no significant difference between the prevalence of hypodontia in males (4.07%) and females (4.89%). Females presented a higher prevalence of congenital missing teeth, which is in agreement with the majority of reports by Grahnèn [24], Haavikko [25], Fekonja [6], and Endo et al. [8]. But Larmour et al. [26] found that in the primary dentition, there was no gender distribution, while in the permanent dentition, females are affected more frequently than males by a ratio of 3:2. In the study of Behr et al. on the German population [20] and of Laganà et al. on a non-orthodontic Italian sample [27], the percentage was equally distributed between males and females.

Usually, a patient’s first visit to a dental clinic for an orthodontic evaluation occurred between the ages of 9 and 12. However, patients at the same chronological age can have significant differences in mineralization stages. These major differences in mineralization can be found in the lower second premolar buds [28–32].

To prevent a false-positive diagnosis of agenesis, we selected 9 as the minimum age because calcification of teeth has usually not been completed by this age [1], and to prevent classification of late mineralized teeth as congenitally missing, final longitudinal panoramic views were also used to confirm a diagnosis of hypodontia.

In the present study, of the individuals identified with congenitally missing teeth, 84% had one or two missing teeth; this is in accordance with other studies by Davis [33], Fekonja [6], Gomes et al. [34], and Goya et al. [35]. Thus, most of the affected individuals suffer only a mild form of hypodontia.

We found that the most often congenital missing tooth types in patients observed in our study were mandibular second premolars, followed by maxillary lateral incisors and maxillary second premolars. Lo Muzio et al. [14] and Laganà et al. [27] had similar findings in the previous study on the Italian population, whereas Polastri [15] found that the most affected tooth was the maxillary lateral incisor followed by the mandibular second premolar.

There is some variation in the literature concerning the description of the most frequently missing tooth, excluding third molars. In the European population, the teeth that were most frequently affected by hypodontia are the following: mandibular second premolar, maxillary lateral incisor, and maxillary second premolar [10]. The mandibular second premolar is the most frequently missing tooth also reported by Polder et al. [10], Endo et al. [8], Tunc et al. [36], Goya et al. [35], and Kirzioglu et al. [37]. In Malaysian [19], Turkish [35], and American populations, the most commonly missing tooth was the maxillary lateral incisor [13]. In the Chinese population, the most frequently missing teeth are mandibular central and lateral incisors [10]. Teeth with the lowest frequency of agenesis were canines (6 males and 15 females) and the first molars (0 males and 3 females). The first molar was missing only in patients with oligodontia.

We found more missing teeth in the maxilla than in the mandible, and the difference was not significant. This result corresponds with the analysis performed by Peker et al. [38], as well as Fekonja [6] and Wong et al. [39] who found missing teeth considerably more frequently in the upper arch than in the lower arch in orthodontic patients. However, Kirzioglu [37] found more missing teeth in the mandible than in the maxilla. Gomes et al. [34] found maxillary hypodontia in 59.2% of patients and in the mandible of 40.8% with an overall ratio of 1.45:1 in orthodontic patients.

Bilateral agenesis manifested a frequency of 4.4%. The most common bilaterally missing teeth were the mandibular second premolar and the maxillary lateral incisor. Goya et al. [35] found that symmetry of congenitally missing teeth was predominant (74.6%), and Kirzioglu et al. [37] observed that bilaterally missing teeth was 73.2%. Moreover, symmetrical hypodontia was predominant, being found in both the contralateral and antagonistic quadrant, possibly suggesting a strong genetic pattern of hypodontia. It was demonstrated also that permanent tooth agenesis, maxillary lateral incisor microdontia, palatally displaced canines, and distoangulation of mandibular second premolars were frequently associated with maxillary lateral incisor agenesis, providing additional evidence of a genetic interrelationship in the causes of hypodontia [40]. Moreover, a significant decrease in maxillary transversal and sagittal size was demonstrated in patients with dental agenesis [41].

## Conclusions

We found a higher prevalence of congenital missing teeth (9%) compared to previous similar studies, so hypodontia is not an uncommon anomaly in the Italian population. There were no significant differences in the distribution of congenitally missing teeth between the sexes or in localization by arches and quadrant sides. The mandibular second premolars were the most frequently missing teeth, followed by the maxillary lateral incisors and maxillary second premolars. By early detection of missing teeth, alternative treatments can be discussed and planned with a multidisciplinary team to minimize the complications of congenital missing teeth and to restore the patient’s dental esthetics and functionality.
